# The Importance of Shiga Toxin-Producing *Escherichia coli* O145:NM[H28]/H28 Infections in Argentina, 1998–2020

**DOI:** 10.3390/microorganisms10030582

**Published:** 2022-03-07

**Authors:** Claudia Carolina Carbonari, Elizabeth Sandra Miliwebsky, Gisela Zolezzi, Natalia Lorena Deza, Nahuel Fittipaldi, Eduardo Manfredi, Ariela Baschkier, Beatriz Alejandra D’Astek, Roberto Gustavo Melano, Carla Schesi, Marta Rivas, Isabel Chinen

**Affiliations:** 1 Servicio Fisiopatogenia, Departamento Bacteriología, Instituto Nacional de Enfermedades Infecciosas-ANLIS “Dr. Carlos G. Malbrán”, Buenos Aires 1282, Argentina; emiliwebsky@anlis.gob.ar (E.S.M.); gzolezzi@anlis.gob.ar (G.Z.); ndeza@anlis.gob.ar (N.L.D.); emanfredi@anlis.gob.ar (E.M.); abaschkier@anlis.gob.ar (A.B.); badastek@gmail.com (B.A.D.); cschesi@anlis.gob.ar (C.S.); mrivas@inmunova.com (M.R.); ichinen@anlis.gob.ar (I.C.); 2 Faculty of Veterinary Medicine, University of Montreal, Saint-Hyacinthe, QC J2S 2M2, Canada; nfittipaldi@umontreal.ca; 3 Public Health Ontario, Toronto Laboratories, Toronto, ON M5G 1M1, Canada; roberto.melano@oahpp.ca; 4 Department of Laboratory Medicine and Pathobiology, University of Toronto, Toronto, ON M5G 1M1, Canada

**Keywords:** STEC O145, hemolytic uremic syndrome, genetic diversity, epidemiology, surveillance

## Abstract

Shiga toxin-producing *Escherichia coli* (STEC) is known as a pathogen associated with food-borne diseases. The STEC O145 serogroup has been related with acute watery diarrhea, bloody diarrhea, hemorrhagic colitis, and hemolytic uremic syndrome (HUS). Argentina has the highest rate of HUS worldwide with 70% of the cases associated with STEC infections. We aimed to describe the epidemiology and genetic diversity of STEC O145 strains isolated across Argentina between 1998–2020. The strains isolated from 543 cases of human disease and four cattle, were pheno-genotipically characterized. Sequencing of five strains was performed. The strains were serotyped as O145:NM[H28]/H28, O145:H25, and O145:HNT, and mainly characterized as O145:NM[H28]/*stx*_2a_/*eae*/*ehxA* (98.1%). The results obtained by sequencing were consistent with those obtained by traditional methods and additional genes involved in different mechanisms of the pathogen were observed. In this study, we confirmed that STEC O145 strains are the second serogroup after O157 and represent 20.3% of HUS cases in Argentina. The frequency of STEC O145 and other significant serogroups is of utmost importance for public health in the country. This study encourages the improvement of the surveillance system to prevent severe cases of human disease.

## 1. Introduction

Shiga toxin-producing *Escherichia coli* (STEC) was first described as a food-borne pathogen in 1982, associated to two outbreaks of hemorrhagic colitis [1]. STEC infections range from mild to severe diseases. The cases, sporadic or associated to outbreaks, are typically related to the consumption of contaminated food or water [2]. Oral fecal transmission (person to person and animal to person) is also a frequent route of infection. Ruminants, especially cattle, are recognized as a STEC reservoir [3].

Virulent STEC strains are characterized by the production of Shiga toxin (Stx). Two types of Stx are described as Stx1 and Stx2, and classified into different subtypes. Additionally, STEC shows other pathogenicity-associated genes, the intimin, encoded by the *eae* gene in the Locus of Enterocyte Effacement (LEE) pathogenicity island, and enterohemolysin gene (*ehxA*) carried in the large virulence plasmid [4].

In addition to the most studied O157:H7 prototype, other non-O157 STEC, such as O26, O111, O145, O103, and O121, have also emerged and became a significant public health problem worldwide. Particularly, STEC belonging to the O145 serogroup, including O145:NM, O145:H28, O145:H25, O145:H34, O145:H8, O145:H16, and O145:HNT, have been associated with acute watery diarrhea (D), bloody diarrhea (BD), hemorrhagic colitis, and hemolytic uremic syndrome (HUS) [5,6].

Argentina has the highest rate of HUS worldwide. In 2019, the estimated annual incidence rate of HUS reported to the Public Health System was 7.25 cases per 100,000 children aged under five years old. It was estimated that approximately 70% of HUS cases were associated with STEC infections, O157 being the predominant serogroup related to severe disease [7]. In a case-control study of Argentinian children with bloody and non-bloody diarrhea and HUS, it was shown that the most common STEC serotypes, after O157:H7, were O145:NM (12.6%), O26:H11 (5.8%), O113:H21 (3.9%), O174:H21 (2.9%), and others (15.6%) [8]. During the STEC risk factor study conducted from 2004 to 2010, O157:H7 was the predominant (>70%), and O145:NM (13.6%) the second most important identified [9] type, among the multiple serotypes detected.

Considering STEC O145:NM[H28] as the second most frequent serotype associated to HUS in Argentina over time, we aimed to describe the epidemiology and genetic diversity of this pathogen related to human infections between 1998 and 2020. In addition, the analysis of the results from the first sequences of STEC O145 strains isolated from human cases is shown as an initial approach to a better understanding of their genomic content.

## 2. Methods

### 2.1. Study Design

This is a retrospective and descriptive study. For the analysis, demographic and epidemiological data from cases and bacteria characteristics were considered. The data related to surveillance and outbreaks investigation were collected from National Health Surveillance System (SNVS 2.0) through the Integrated System of Argentine Sanitary Information (SISA) platform.

Briefly, the SNVS-surveillance was implemented considering the high incidence of HUS in the country through the mandatory report of individual cases (Resolution 346/2000, Ministry of Health). In this context, the implementation of a specific protocol for prevention and control strategies was mainly applied in closed communities, such as home and institutional environments. In addition, the notification of the index case to the public health system is the starting-point of a series of measures, such as: (1) Control measures implementation for monitoring of the clinical evolution and optimization of the hygienic and sanitary condition for the contact group; (2) Identification of possible vehicles and sources by epidemiological survey; (3) Detection of possible symptomatic or asymptomatic STEC infections through a cohort study in the family and/or institutional closed contacts; and (4) Long shedding follow-up of patients and positive contacts to diminish the transmission. Moreover, it was established that STEC positive cases are not able to the day-care centers, kindergarten, or school, until they are negative in two successive sampling controls.

Furthermore, this procedure provides the system the ability to detect small outbreaks that may be ongoing in these closed communities. An outbreak event was defined as more than one STEC-associated case occurring at the same time in a closed community/family.

### 2.2. Bacterial Isolates

A total of 581 STEC O145 strains isolated from humans (*n* = 577) and bovines (*n* = 4) in Argentina between 1998 and 2020 were selected from the National Reference Laboratory (NRL) collection for this study. The human strains were obtained from samples or cultures submitted to the NRL for further characterization, through the National Diarrhea and HUS Laboratory Network. The four STEC O145 strains were isolated from fecal or carcass samples of beef cattle, during animal monitoring or research projects. The results of adhesins and eae characterization from a subset of 286 strains isolated during 1998–2012 (already included in the 581 strains), obtained in the framework of a research project, were included in this analysis.

### 2.3. Phenotypic Characterization

All STEC O145 strains included in this study were identified and pheno-genotypically characterized following procedures previously described [10]. Biochemical identification was performed by conventional methods. Sorbitol fermentation was evaluated using red phenol broth containing 1% D-sorbitol and β-glucuronidase activity was assayed with Coli-Brit discs (Britania Laboratory, Buenos Aires, Argentina). Motility was measured using Cragie media. The organisms were serotyped to detect O serogroups (lipopolysaccharide) and H (flagellar) antigens. Specifically, O antisera produced and provided by Serum and Antigen Service of the INPB-ANLIS “Dr. Carlos G. Malbrán” and Denka Seiken (Japan) flagellar antisera were used. Organisms were cultured on sheep blood agar plates to identify enterohemolysin activity. Susceptibility against amikacin, ampicillin, ciprofloxacin, chloramphenicol, gentamicin, nalidixic acid, norfloxacin, streptomycin, tetracycline, and trimethoprim-sulfamethoxazole were measured by disc diffusion CLSI methods. The presence of β-lactamases and extended spectrum β-lactamase (ESBL) activity were measured using the double disc diffusion method. Susceptibility was inferred using CLSI breakpoints [11].

Multiplex PCR was performed as previously described to identify the Shiga toxin-encoding *stx*_1_ and *stx*_2_ genes [12]. Genotypic confirmation of the O145 serogroup was made following the methods described by Fratamico et al. [13] with minor modifications (0.1mM dNTP, 1.5 mM ClMg, and 0.02 U/μL Taq polymerase). Determination of the presence of the flagellar gene (*fliC*) was carried out using the adapted *fliC*_H28_ and *fliC*_H8_ primer sequences described by Bugarel et al. [14]. The *eae* and *ehxA* genes were determined as previously detailed [15,16]. Moreover, the presence of putative adhesins *lpfA*_O113_ and *lpfA*_1-5_ genes, were determined also by PCR [17] in a subset of 286 strains isolated during 1998–2012.

The identification of *stx*_1_ subtypes was performed according to the methods described by Zhang et al. [18]. Regarding *stx*_2_ subtypes (STEC *eae*_+_)_,_ were detected by PCR-RFLP of the *stx*_2_ gene in comparison to the reference *E. coli* organisms EDL933 O157:H7 (*stx*_1_ and *stx*_2_), E32511 O157:NM (*stx*_2vh-a_) and 93-016 O113:H21 (*stx*_2vh-b_) [19]. The strains isolated from 2018 onwards were determined by the methodology described by Scheutz et al. [20]. All the *stx* subtypes shown in this study were named in accordance to the last nomenclature [20]. Variants of the *eae* gene were determined using primers and conditions described by Ramachandran et al. [21] in a subset of 286 strains isolated during 1998–2012.

Macrorestriction fragment analysis by pulsed-field gel electrophoresis (PFGE) was done using the PulseNet standardized protocol for STEC non-O157 [22]. Images of the resulting PFGE gels were captured using Doc-It 2000 (Bio-Rad, Hercules, CA, USA) and analysed by BioNumerics software package version 5.1 (Applied Maths, Kortrijk, Belgium) using the Dice coefficient and the UPGMA to generate dendrograms with 1.5% tolerance values. The analysis was focused on clusters detection and outbreaks investigation support. A cluster was defined as a group of two or more strains with identical *Xba*I-PFGE patterns.

### 2.4. Sequencing and Analysis

Five strains were selected to perform whole genome sequencing (WGS) in the framework of a pilot project. Four strains from HUS cases and the *stx*_1_ strain isolated from an asymptomatic contact, belonging to different PFGE patterns, were included. The sequencing lab procedure and the genomic analysis were carried out at the Genome Core, Public Health Ontario. Strains were grown overnight on Tryptic soy agar (BD-Difco™, Le Pont de Claix, France), and genomic DNA extraction (QIAamp DNA Mini Kit, Qiagen Group) and quantification (Qubit^®^fluorometer, Invitrogen, Eugene, OR, USA) were performed according to the manufacturer protocols. Libraries were prepared using the Nextera XT DNA Library Preparation Kit (Illumina, San Diego, CA, USA), and sequenced as paired-end reads (150 bp + 150 bp) on an Illumina MiSeq instrument (Illumina 1.9, San Diego, CA, USA).

Quality of raw reads was assessed with FastQC v 0.11.5 [23]. Paired-end reads were de novo assembled using the A5 pipeline [24] and the contigs obtained were reordered against reference strain RM131514 [25] using Mauve v 2.3.1 [26]. The sequences were annotated with the NCBI Prokaryotic Genomes Automatic Annotation Pipeline [27]. Genome assemblies were analysed using tools from the Center for Genomic Epidemiology’s web interfaces: SerotypeFinder 2.0 [28], VirulenceFinder 2.0 [29,30], ResFinder 4.1 (acquired antibiotic resistance genes) [31,32,33], PlasmidFinder 2.1 (in silico detection and typing of plasmids) [34] and MLST 2.0 (Multilocus Sequence Typing of Total Genome Sequenced Bacteria) [35]. In addition, the PHASTER (PHAge Search Tool Enhanced Release) web interface was used for the identification of phages [36].

### 2.5. Statistical Methods

Prevalence of STEC in all samples and prevalence associated with other variables (diagnosis, gender, and places) were calculated with 95% confidence intervals (CIs). Statistical calculations were performed with the Pearson’s chi-square test using the software EPI Info v.7.2.1.0 (CDC, Atlanta, GA, USA, 2016). A *p* value of ≤0.05 was considered statistically significant.

## 3. Results

### 3.1. Epidemiological Features of STEC O145 Infections in Argentina

**Figure 1:**
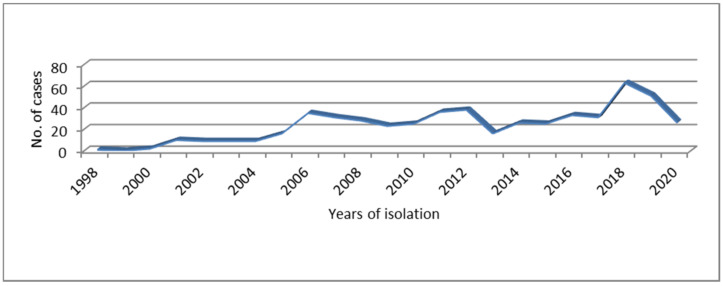
Distribution of Shiga toxin-producing *Escherichia coli* (STEC) O145 infections occurred in Argentina over time.

From clinical samples and cultures received between 1998 and 2020 at the NRL, 3502 STEC strains corresponding to 3368 cases were isolated for further characterization. Out of the total, 577 (16.5%, 95% CI 15.2–17.7), including 34 strains isolated during shedding monitoring, were identified as STEC O145. These strains corresponded to 543 (16.1%, 95% CI 14.9–17.4) cases that occurred across 18 provinces. The higher proportion of the cases belonged to the province of Buenos Aires (199; 36.6%, 95% CI 32.6–40.7) and the city of Buenos Aires (135; 24.9%, 95% CI 21.2–28.5). The annual trend of the cases associated to STEC O145 from 1998 to 2020 (Figure 1) showed variations through the years and ranged from one in 1998 to 63 cases in 2018. A marked increasing number of cases was observed in 2018 in comparison with 2017 but this was not statistically significant (*p* > 0.05).

The NRL recorded the first isolation of STEC O145 in 1998, corresponding to a HUS case notified in the province of Mendoza. The majority of STEC O145 from humans were associated to HUS cases (309/543; 56.9%, 95% CI 52.7–61.1), with five fatal cases (5/309; 1.6%, 95% CI 0.2–3.0). The remaining STEC O145 strains were isolated from bloody diarrhea (100/543; 18.4%, 95% CI 15.1–21.7), non-bloody diarrhea (89/543; 16.4%, 95% CI 13.3–19.5), ulcerative colitis (1/543; 0.2%), asymptomatic contacts of HUS cases (33/543; 6.1%, 95% CI 4.1–8.1), and patients without a known clinical diagnosis (11/543; 2%, 95% CI 0.8–3.2).

**Figure 2:**
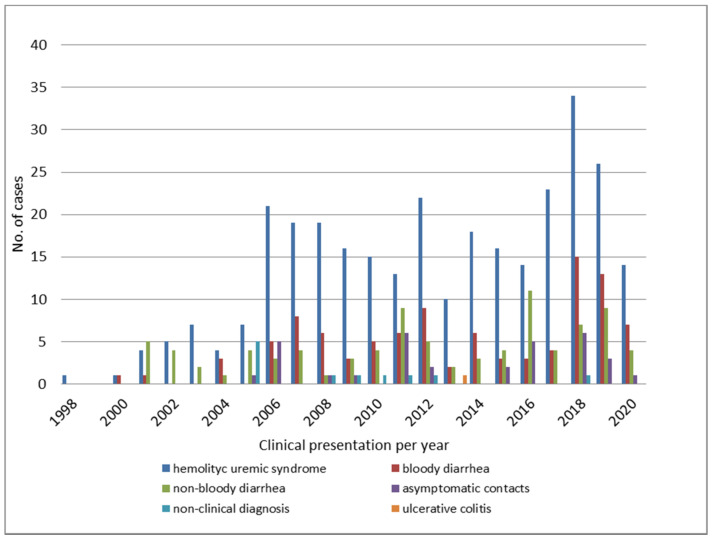
Clinical presentation of STEC O145 infections throughout the years.

The distribution of STEC O145 by year, according to the disease presentations between 1998 and 2020, is shown in Figure 2.

Regarding the frequency of the cases by gender, 53.8% (95% CI 49.6–57.9) and 43.5% (95% CI 39.3–47.6) were female and male, respectively. A small percentage (2.7%) of data was not available.

The four STEC O145:NM[H28] strains of bovine origin were isolated in three different cities from the province of Buenos Aires, in the framework of two different research projects. The first strain of STEC O145 from a bovine reservoir, recorded by the NRL, was isolated in 1999 in the city of Castelar. Further, two strains were recovered from a calf in the city of Tres Arroyos in 2007 and the last one from a young steer in the city of San Pedro in 2008.

In Argentina, STEC is mainly associated with sporadic cases; however, outbreaks are also detected. Twenty-two independent STEC O145 outbreaks (Supplemental Material Table S1) were identified in 16 locations in six different provinces and the City of Buenos Aires (CABA): Buenos Aires (*n* = 7), CABA (*n* = 4), Santa Fe (*n* = 1), Córdoba (*n* = 1), Neuquén (*n* = 1), Río Negro (*n* = 4), and La Pampa (*n* = 4). Seventeen of these outbreaks were detected in family environments, between 2006 and 2020, in different locations. Most of these outbreaks (14/17; 82%) involved one case of HUS/BD and 1–3 asymptomatic contacts. Nevertheless, two outbreaks of HUS cases (3 HUS cases; 1 fatal case and 2 HUS) and one of diarrhea cases (6 diarrhea cases), occurred.

The five outbreaks in day-care centers/kindergartens were detected in four different locations. Four of them involved more than one case of HUS/BD/D and 3 also 1–2 asymptomatic contacts; just one outbreak was described with one HUS case and 2 asymptomatic contacts. Only in one outbreak was found a co-infection case with another serogroup (outbreak 6: O111:NM *stx*_1a_/*eae*/*ehx*A); also 3 cases of BD and D were described as long carriers.

Despite the effort done during the epidemiological investigation to find out the vehicles and sources of the infection, they could not be detected.

### 3.2. Phenotypic and Genotypic Characterization of STEC O145 in Argentina

All 581 strains phenotypically serotyped as O145 were confirmed by PCR amplification. The majority (518/581; 89.2%) of STEC O145 was found to be non-motile (NM). Out of these, 508 (98%) strains were PCR *fli*C_H28_ positive and the remaining 10 (2%) strains were negative for *fli*C_H28_/*fli*C_H8_. The motile isolates (63/581; 10.8%) were further characterized as O145:H28 (55/63; 87.3%), O145:H25 (3/63; 4.8%), and O145:HNT (5/63; 7.9%) serotypes. Additionally, 578/581 (99.5%) of the strains had the ability to ferment sorbitol and 472/581 (82%) exhibited β-glucuronidase activity.

Most of the STEC O145 strains were characterized as *stx*_2a_ (580/581; 99.8%) and *stx*_1a_ (1/581; 0.2%) by *stx* subtyping. The presence of the *eae* and *ehxA* genes, was detected in all of the screened isolates with the exception of eleven (11/581; 1.9%), which were negative for *ehxA* either by PCR or phenotypic assay results.

Out of the total, 286 (from 1998–2012 period) strains were subtyped for the *eae* gene and *lpfA*_1-5_ and *lpfA*_O113_ adhesins genes. These strains belonged mainly to *eae*-γ (279/286; 97.6%) and seven (2.4%) harboured *eae*-β variants. Regarding the adhesins, the majority of organisms carried the *lpfA*_1-5_ gene (275/286, 96.2%), and a limited number the *lpfA*_O113_ gene (15/286; 5.2%).

By antimicrobial susceptibility testing, it was found that most of the organisms showed pan-susceptibility to the selected antimicrobials, with only 50/581 (8.6%) STEC O145:NM being resistant to at least one of the antimicrobials tried. We identified 17 unique antimicrobial resistance profiles and seven isolates exhibited resistance to four of the antimicrobials tested (Table 1). Whilst they were not screened for phenotypic susceptibility against third generation cephalosporins, the organisms that were resistant to ampicillin (*n* = 16) were selected for further β-lactamase and ESBL activity investigation using amoxicillin-clavulanic acid, ceftazidime, cefotaxime, cefoxitin, and cephalothin. Despite β-lactamase activity being detected, the mechanism of antibiotic resistance was not observed.

### 3.3. Molecular Epidemiology of Argentinian STEC O145

A total of 581 STEC O145 strains were performed by *Xba*I-PFGE, generating 402 different restriction patterns (with at least 60.1% similarity). Two hundred and thirty three strains were grouped into 73 clusters (named as No.1–No.73), 329 strains exhibited unique *Xba*I-PFGE patterns, and nineteen strains were non-typeable. A dendrogram with a representative of each pattern is included as Supplemental Material (Figure S1); the distribution of the clusters and outbreaks are also indicated in the figure. The frequency of the prevalent *Xba*I-patterns corresponding to nine clusters were: ARENMX01.0006 (13 strains; 3.3%), ARENMX01.0002 (11; 2.7%), ARENMX01.0004/0076/0084/0120 patterns (8; 2.0%, each), ARENMX.0281 (7; 1.7%); and ARENMX.0303/0061 (6; 1.5%, each). The rest of the clusters (*n* = 64) contained less than five strains.

**Figure 3:**
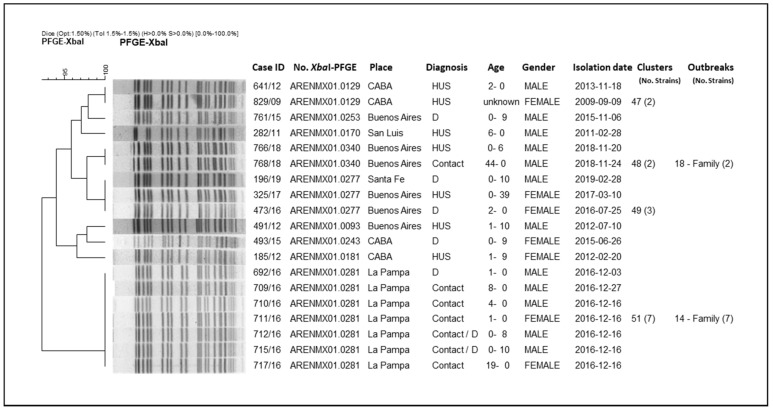
Clonal relatedness of different STEC O145 strains by *Xba*I-PFGE. City of Buenos Aires (CABA).

**Figure 4:**
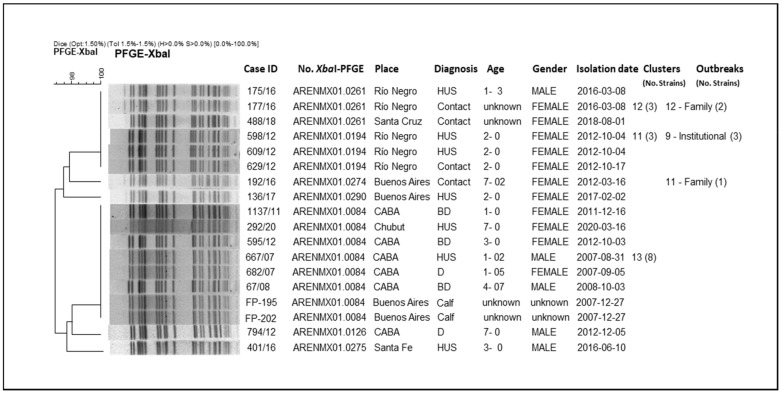
Clonal relatedness of different STEC O145 including one cluster with human and bovine origin strains, by *Xba*I-PFGE.

From the analysis of the clusters, we could observe that they mostly grouped strains isolated at different times and locations across the country. Some outbreaks included: strains that grouped in clusters; strains with high-related patterns (not identical); and strains corresponding to both cluster and related patterns. In order to present the results of the clonal relationship among the strains, the analysis of a two particular branch of the whole tree (*n* = 562) are shown as an example (Figure 3 and Figure 4).

In Figure 3, it is shown how these strains are grouped into an entire branch, presenting patterns with at least more than 93% similarity; they correspond to four clusters and five unique patterns. The clusters are: No. 51 (pattern ARENMX01.0281; 7 strains); No. 48 (pattern ARENMX01.0340; 2 strains); No. 49 (pattern ARENMX01.0277; 3 strains); and No. 47 (pattern ARENMX01.0129; 2 strains). The cluster No. 51 and No. 48 correspond to family outbreaks. The first includes the index case and six family contacts with diarrhea (outbreak No. 14/La Pampa, 2016). The second one corresponds to two strains isolated from an HUS case and his father, an asymptomatic contact (outbreak No. 18/Buenos Aires, 2018). Cluster No. 47 includes two HUS cases (occurred at the same location and different times), and cluster No. 49 presents one HUS and two diarrhea cases (different place and time). The five unique patterns were detected once in the entire period of study.

In Figure 4, the strains are grouped with more than 96% similarity. Only one cluster (No. 13) contains both human and bovine isolates with 100% identity pattern (ARENMX01.0084; 8 strains). The rest of the clusters in the whole tree, grouped exclusively human strains, such as Cluster No. 12 (pattern ARENMX01.0261; 3 strains) and No.11 (pattern ARENMX01.0194; 3 strains), correspond to family and institutional outbreaks, respectively. The first includes a HUS case and his mother (outbreak No. 12/Río Negro, 2016). For the second, two HUS cases and one contact were involved (outbreak No. 9/Río Negro, 2012). Both clusters (No. 12 and 11) are almost identical with just one band difference; these clusters could not be discriminated by BioNumerics due the tolerance parameters for analysis. In addition, four strains present unique patterns (Supplementary Material Figure S1–Table S1).

Regarding the total outbreaks (Table S1), previously described, they were associated with strains of different PFGE patterns and two outbreaks presented the most frequent pattern (ARENMX01.0006).

### 3.4. Sequencing and Genomic Analysis

The Whole Genome Shotgun project has been deposited at DDBJ/ENA/GenBank under the accession PRJNA769431. The versions described in this paper are the first draft genome sequences of these five STEC O145:NM, under the accession number listed in Table 2.

The strains were confirmed to be of O145 serogroup by SerotypeFinder using default parameters (select threshold for % ID = 85%, and select minimum length = 60%) and *fliC*_H28_ was identified in four genomes. The one, which the flagellar *fliC*_H28_ gene was not identified in the genome, was positive by *fliC*_H28_ end-point PCR.

Regarding MLST, all STEC O145 strains belonged to ST-32.

VirulenceFinder was run using default parameters (select threshold for % ID = 90%, and select minimum length = 60%). Out of the five strains sequenced, four were positive for *stx*_2a_ and only one strain for *stx*_1a_. All the strains carried *eae* gene (intimin) and four the *ehxA* gene. In addition, other genes such as *tir*, *espA* and *espB* (LEE-encoded type III secretory system proteins), *iha* (IrgA homologue adhesin), and *nleA*, *nleB*, and *nleC* (non-LEE encoded effector protein encoding genes) were present. Furthermore, phage-encoded type III secretory system protein encoding genes such as *espI*, *espJ*, and *cif* were detected as well. Regarding other plasmid-encoded virulence genes *katP* and *espP* were found in all*,* except in one strain, in which the *katP* gene was not identified. Others, such as the *astA*, *chuA*, *iss*, *iucC*, *iutA*, *neuC*, *ompT*, *terC*, and *traT* genes, were present in all strains. *Tox*B was found in three strains and only one strain carried *cma*, *cvaC*, *hlyF*, *iroN*, and *mchF* genes.

On the other hand, the *mdfA* antimicrobial resistance gene was found in all the strains using ResFinder with default parameters (select threshold for % ID = 90%, and select minimum length = 60%). Two strains also presented the *pmrB* gene associated with Colistin resistance and one strain presented *bla*TEM-1B, *aadA1*, *dfrA1, tetA*, *sul1*, and *sul2*.

The virulence and resistance genes’ content is provided in Table 3 in more detail.

From the detection and typing, the most common sequences of plasmid found in all the STEC O145 strains were IncB/O/K/Z and IncFIB (AP001918). In addition, IncX4 was present in one strain. Moreover, another strain carried IncFII and IncQ1 plasmids as well.

Finally, the average number of phage sequences was 4.8 (3–7), considering intact, incomplete, and questionable phages based on PHASTER scores. The most common phage sequences found among these strains are shown in Table 4. The intact phage sequences found were Yersin_L_413C_NC_004745, Entero_mEp460_NC_019716, and Entero_P2_NC_001895.

## 4. Discussion

Currently, technological advances in detection methodologies have made the surveillance and notifications, mainly for STEC non-O157, possible in different countries. The frequency of association to human illness according to the different serogroups varies worldwide. In Argentina, STEC O145 strains are of utmost importance, being the second serogroup after O157. They were detected in 543 cases, representing 16.1% of the STEC strains isolated from clinical samples. According to the NRL, between 1998 and 2020, the proportion of cases associated to prevalent serogroups was: STEC O157, 71.9% (*n* = 2423/3368); STEC O26, 2.4% (*n* = 80/3368); STEC O121, 1.8% (*n* = 60/3368), and STEC O103, 1.1% (*n* = 36/3368). In comparison, in the United States it was described that STEC O26 (22%) is the second serogroup associated with human illness after STEC O157:H7; STEC O145:NM represents a small percentage (5%) which ranks sixth place among detected serogroups [37,38]. In Europe, EHEC O26:H11/H− accounted for 5–7% of all human EHEC isolates [39]. Moreover, in Germany, O26:H11/H-(13%) was the most prevalent non-O157 STEC isolated, followed by O103 (5%) and O145 (4%). These results agree with the prevalence data reported in other countries of Continental Europe, including Austria, Belgium, the Czech Republic, Finland, France, Italy, Serbia, Switzerland, and the Netherlands, in patients with severe disease such as HUS and also uncomplicated diarrhea [40].

Out of the total cases associated with STEC O145 during this period, 56.9% corresponded to HUS cases and five of them were deceased (1.6%), which demonstrates the association of STEC O145 with severe disease in Argentina. The HUS-STEC O145 (*n* = 309) represents 20.3% of all the HUS cases (*n* = 1521) in the same period, confirming that it is the second serogroup associated with severe disease in our country after STEC O157:H7 (69.3% STEC O157 associated HUS). In third place, STEC O121 was found with 2.4%, showing a great difference in frequency with the main serogroups. This is available in the national reports [7,41] where STEC O157:H7 and O145:NM/H28 were detected through the years. Apart from the association with severe disease, this pathogen was detected in bloody diarrhea (18.4%), diarrhea (16.4%), asymptomatic contacts (6.1%), unknown clinical diagnosis (2%), and ulcerative colitis (0.2%). Increasing detection was observed along the years, from one strain in 1998 to sixty-one in 2018, with a higher percentage of HUS associated with O145 detected during 2018–2019. This could be related to an improvement in the surveillance system and the methodological diagnosis implemented for STEC non-O157. For instance, Oderiz et al. [42] has reported the local detection of O145:NM (16.5%) in samples processed in a specialized hospital in the city of La Plata, province of Buenos Aires. It is estimated that this pathogen has been circulating in our country since several years ago, considering that the first isolate was detected from a HUS case notified in 1998, at the moment the NRL started to implement specific *stx*-PCRs [43].

Despite most of the cases being reported as sporadic events, outbreaks associated to STEC O145 were detected. The surveillance system strategy implemented in the country enables the possibility of detecting closed community outbreaks. In general, the events occur in familiar or institutional environments. During this study, the number of persons involved in each outbreak detected by the epidemiological follow-up procedure showed that person-to-person transmission was the most likely route of dissemination. Regardless of the effort in the investigation, the source association could not be identified; nevertheless, one of the kindergarten outbreaks was highly associated with recreational water, even though no STEC strain was recovered to confirm this hypothesis. Other countries have also reported O145-associated outbreaks. In Germany, an outbreak that occurred in 1999 has been described as two STEC O145:H28 positive ill persons, without association to the source [38]. In Belgium, a STEC O145 outbreak was reported associated to ice cream as vehicle of infection. This event occurred in two successive birthday parties celebrated in a farm with five HUS cases; four of them were confirmed as STEC O145 positive and one included a co-infection with STEC O26 [44]. In 2009, in Norway an outbreak in a day-care center with 16 diarrhea cases, particularly associated to STEC O145:H28/*stx*_1_/*eae*, was notified [45]. In the United States, a multistate outbreak was reported in 2010, associated with lettuce consumption with 26 confirmed (three HUS) cases from five states [46]. During 2012, another one with 18 STEC O145 cases was notified from nine different states (four hospitalized patients and one of them deceased), without source confirmation [47].

The surveillance system strategy has also allowed the long shedding study of the cases and contacts. In total, 12 cases with more than one positive sample identified during fecal shedding were detected (data non-shown). The outbreak occurred in Rosario with a O145:NM-positive boy being a clear example of intermittent and persistent shedding over time, around 20 days [48]. The long-term shedding of STEC has been previously described in Germany [49].

The low infective dose of STEC is a determining factor which facilitates the pathogen’s transmission, mainly person to person and through food consumption [43]. In addition, there are several host factors that influence the risk of acquiring STEC infection, including age, immunity, health status, the use of antibiotics and antimotility agents, stress, and genetic factors [9]. Therefore, it is always important to perform follow up of the patients and evaluate the STEC infections among cohabitants as well, to interrupt the cycle of transmission.

In this study we described STEC O145:NM strains from bovine, but it is not a frequent serotype isolated from animals in Argentina. Three O145:NM strains of bovine origin included in this study were isolated in the framework of an investigation done in abattoirs, representing only 1% of the strains isolated from bovine feces [50]. The other one corresponded to a study in collaboration with INTA during 1999 [51]. Other authors described not having identified O145 among the 31 serogroups detected in carcasses, cuts, and trimming in an exporting abattoir in the country [52]. In a beef slaughterhouse that supplies the domestic market in Argentina, STEC O145 was detected in hides (4/22; 19%) and carcasses (1/27; 4%), even though the strains could not be isolated [53].

In our country, STEC O145 has not been found in food so far; nevertheless, it is important to reinforce the surveillance in food, considering that this pathogen has been associated with illness after the consumption of different types of food (i.e., ice cream and lettuce) [44,46], and also of undercooked venison meat in the United States [54].

In this study, the majority of STEC O145 human strains (*n* = 577) were O145:NM (89.1%); however, it was possible to determine the *fliC*_H28_ and were genotyped as O145:NM[H28] (87.3%). The motile strains were detected as O145:H28, O145:H25, and O145:HNT (9.5%, 0.5%, and 0.9% respectively). These serotypes are the most commonly described for STEC O145 by different authors [6,55]; as they are mostly non-motile strains, the *fli*C detection is essential for full characterization. Moreover, variants of the flagellar antigen genes of O145 were also described [56].

All strains were characterized as *stx*_2a_ (*n* = 576), except only one, *stx*_1a_, associated to an asymptomatic contact. A high frequency of STEC O145-*stx*_2a_ is detected in agreement with the results described in other countries; additionally, also other strains with both *stx*_1a_ and *stx*_2a_ were reported in less proportion [37,57,58]. Furthermore, *stx*_1a_ strains were also associated with an outbreak of diarrhea in Norway, as it was mentioned before [45].

Most of the strains were characterized as *eae* and *ehxA* positive, except eleven that were negative for *ehxA*. Regarding *eae* subtypes, the association of STEC O145:NM[H28]/H28 and O145:H25 to *eae*-γ and *eae*-β variants was respectively observed; as it was described by Sonntag [6]. STEC strains with different *fliC* such as O145:H34 *stx*_2f_ codifying *eae-*ι variant were associated to acute diarrhea in Brasil [59].

The majority of organisms carried the *lpfA1-5* gene (96.2%), and a limited number contained the adhesin *lpfAO113* gene (5.2%). This association between *lpfA1-5* gene and STEC O145 was previously described [60]. The genes encoding fimbrial structures are usually present in pathogenic *E. coli* strains and, especially, *lpfA1* and *lpfA2* genes are frequently found among LEE-positive strains associated with severe disease [61].

In summary, the prevalent virulence profile was O145:NM[H28]*/stx*_2a_*/eae/ehxA* (98%; 569/581) among human strains. Additionally, considering the results of adhesins analysed in the subset, the prevalent profile was O145:NM[H28]*/stx*_2a_*/eae*-γ*/ehxA/lpfA1-5* (90%; 258/286). The O145:NM[H28]*/stx*_1a_*/eae*-γ*/ehxA/lpfA1-5* profile was detected in a unique strain.

Respecting the four strains from bovine origin, all were characterized as STEC O145:NM[H28] *stx*_2a/_*eae*-γ*/ehxA/lpfA1-5*, same feature profile like most of the strains from human origin. Likewise, STEC O145*/stx*_1_ and/or *stx*_2_*/eae/ehxA* strains were isolated from cattle according to Cernichiaro study [62]. In research undetaken in a production region in the USA, variations in the expression of virulence traits was observed among the STEC O145:NM isolated from human, animals, water, and wildlife, even though they were genotypically related [63].

By antimicrobial susceptibility testing, we found that the majority of organisms were pan-susceptibility to the antimicrobials selected, as described for STEC O157:H7 [64]. However, 50/581 (8.6%) of STEC O145:NM[H28] were resistant to at least one of the antimicrobials tried; and ESBL was not detected. From the 17 unique antimicrobial resistance profiles, seven exhibited multidrug-resistance to four antimicrobials. Recent reports indicate that antimicrobial-resistant STEC O145 strains are emerging and associated with sporadic and outbreak cases. Multidrug-resistant strains (chloramphenicol, nalidixic acid, streptomycin, sulfamethoxazole, and tetracycline), isolated from an outbreak associated with lettuce, were described [65]. Additionally, during an investigation in the USA, a high prevalence of resistance (*n* = 5; 80%) was observed, azithromycin being the most common, and also 20% of these showed multidrug-resistance [66]. In our study, all STEC O145 from bovine origin were susceptible, in opposition to the results described during research conducted in Scottish beef cattle that showed resistance profiles to ampicillin, tetracycline, streptomycin, and trimethoprim–sulfamethoxazole [67]. These results were similar to those found in our human strains. Beier also described 63% of resistant bovine strains (*n* = 11) with different resistance profiles and 9% of multidrug-resistances, azithromycin being the most common one. It is important to follow up the monitoring of the resistance profile in the surveillance of STEC strains, because the resistant determinant can be acquired by plasmid transmission between different enteric bacteria [66]. It is also considered that some kinds of plasmids have a broad host range and have been found in members of *enterobacteriacceae* from food, animal, and clinical samples. Moreover, monitoring, molecular, and phylogenetic studies will allow a better understanding of infection sources [68] and the role of animal reservoirs or animal-derived foodstuffs in causing human non-O157 STEC infections [69].

In this study, the high diversity of the STEC O145 strains circulating in the country was shown. Among 581 strains, 402 *Xba*I-PFGE patterns were identified. The ARENMX01.0006 pattern was the most frequently detected in Argentina over time (13/402; 3.3%). The patterns associated to outbreaks were variable (Table S1). Regarding cluster No. 13, the same *Xba*I-PFGE pattern was found in strains from both humans and bovines; apparently this pattern was first recognized 2007 and continued circulating intermittently until 2020. Similar association was observed respecting STEC O157:H7 in Argentina during a study where human and bovine strains from the same period were compared, and five clusters were detected with the presence of both [70].

Previous studies have presented STEC O145 sequences; however, this is the first time the draft sequences of O145 from human origin are shown in Argentina. The WGS results of the relevant virulence genes for diagnosis were supported with those obtained by traditional methodologies used in the NRL for STEC surveillance. Just two discrepancies were observed with respect *fliC*_H28_ and *ehxA* genes in two different strains. Two strains, one positive for *fliC*_H28_ and other for *ehxA* by end-PCRs, could not be characterized by sequencing. This might be due to the short paired-end sequences having low mapping coverage of these genes. Regarding the MLST, we found all STEC O145 strains belonged to ST-32.

WGS was described as a reliable and robust one-step process to be applied for STEC diagnostic and characterization. Additionally to the common virulence profile used for routine procedures, a set of genes involved in different mechanisms (LEE-encoded type III secretory system proteins involved in the formation of attachment and effacement lesions in host epithelial cells, non-LEE encoded effector protein encoding genes, phage-encoded type III secretory system protein, and other plasmid-encoded virulence genes) could be simultaneously detected. Regarding antimicrobial resistance, some genes such as *pmrB*, *aadA*1, *dfrA*1, and blaTEM-1B were found, but the presence of *pmrB* gen and blaTEM-1B had not been phenotypically confirmed. The most common sequences of plasmid found in all STEC O145 strains were IncB/O/K/Z and IncFIB. Finally, only three intact phage sequences were found (Yersin_L_413C_NC_004745, Entero_mEp460_NC_019716, and Entero_P2_NC_001895). In a study with the analysis of both human and bovine draft genomes, similar results regarding virulence and resistant genes, phages, and plasmid content were described [71].

This is our first approach to studying STEC O145 genomes; as such, further analysis is needed to improve genome characterization. Up-to-date, these results enable us to know more details about the content of the STEC O145 strains that circulate in Argentina. In addition, since the serotyping is not easy at early screening stages and molecular methods are essential for STEC diagnostics, genomic sequencing is shown to be an option to facilitate routine diagnostics. In this context, WGS implementation will be a great advancement, contributing to the surveillance strategy and also explaining the survival and evolution mechanisms of the STEC O145 population in the country.

The detection of non-O157 STEC remains a challenge for human infection diagnosis. Additionally, monitoring the prevalence of STEC O145 and other significant serogroups is of utmost importance for public health. As the association with severe disease was highlighted in this study, it is essential to strengthen the surveillance system to prevent human cases associated with STEC O145 and other non-O157 STEC in our country.

## Figures and Tables

**Table 1:** Resistance profiles associated to the different clinical presentations.

#	Resistance Profile *	Origin	No Strains
1	AMP	3HUS/2BD	5
2	AMP/CHL/S/TE	HUS	1
3	AMP/S	HUS	2
4	AMP/S/TE	HUS/unknown	2
5	AMP/S/TE/TMS	HUS	5
6	AMP/S/TMS	3HUS/2BD	7
7	AMP/TE/TMS	fatal HUS	1
8	A/TMS	HUS/D	2
9	CHL/S/TE/TMS	HUS	1
10	CHL/TE/TMS	2HUS/D	3
11	G/S/TE	HUS	1
12	NA/T	HUS/BD	2
13	S/TE	HUS/BD	3
14	S/TE/TMS	unknown	1
15	S	HUS	1
16	TE	5HUS/BD/4D	10
17	TE/TMS	HUS/BD/D	3

* Ampicillin (AMP), chloramphenicol (CHL), gentamicin (GE), nalidixic acid (NA), streptomycin (ST), tetracycline (TE) and trimethoprim-sulfamethoxazole (TMS). Hemolytic Uremic Syndrome (HUS), bloody diarrhea (BD) and diarrhea (D).

**Table 2:** Accession numbers and assembly metrics of the annotated STEC O145:NM[H28].

Isolate No.	Origin	Place-Province/ Year of Isolation	NCBI Accession No.	No. of Scaffolds	Genome Size (bp)	*N*_50_	G+C Content (%)
GN1228	*Homo sapiens*	Santa Fe/2002	JAJBBW000000000	123	5,239,290	173,877	50
GN1229	*Homo sapiens*	City of Buenos Aires/2006	JAJBBV000000000	144	5,345,892	158,581	50
GN1230	*Homo sapiens*	Buenos Aires/2007	JAJBBU000000000	156	5,251,985	160,369	50
GN1231	*Homo sapiens*	Neuquén/2007	JAJBBT000000000	127	5,376,704	152,017	50
GN1232	*Homo sapiens*	Rio Negro/2008	JAJBBS000000000	142	5,259,376	201,633	50

**Table 3:** Distribution of virulence and resistance genes in STEC O145 strains.

Virulence Genes	Product	STEC O145 Strains 1228 1229 1230 1231 1232				
Shiga Toxins						
*stx*_1a_	Shiga toxin 1 subtype a					1
*stx*_2a_	Shiga toxin 2 subtype a	1	1	1	1	
Adhesins						
*eae*	Intimin	1	1	1	1	1
*Iha*	IrgA homologue adhesin	1	1	1	1	1
LEE encoded Type III secretory system proteins						
*Tir*	Translocated intimin receptor	1	1	1	1	
*espA*	EPEC secreted protein A	1	1	1	1	1
*espB*	EPEC secreted protein B	1	1	1	1	1
Non-LEE encoded effector proteins						
*nleA*	Non-LEE encoded effector protein A	1	1	1	1	1
*nleB*	Non-LEE encoded effector protein B	1	1	1	1	1
*nleC*	Non-LEE encoded effector protein C	1	1	1	1	
Phage encoded type III secretory system proteins						
*espI*	*E. coli*-secreted protein I	1	1	1	1	1
*espJ*	*E. coli*-secreted protein J	1	1	1	1	1
*Cif*	Cell-cycle inhibiting factor	1	1	1	1	1
Plasmid encoded virulence factors						
*ehxA*	Enterohemolysin	1	1	1		1
*katP*	Catalase peroxidase	1	1	1		1
*espP*	Extracellular serine protease	1	1	1		1
*iroN*	Enterobactin siderophore receptor protein				1	
*iucC*	Aerobactin synthetase	1	1	1	1	1
*iutA*	Ferric aerobactin receptor	1	1	1	1	1
*cma*	Colicin M				1	
*cvaC*	Colicin V (Microcin)				1	
Antimicrobial resistance genes						
*mdfA*	Multidrug transporter	1	1	1	1	1
*aadA1*	Aminoglycoside resistance				1	
*dfrA1*	Trimethoprim resistance				1	
*pmrB*	Colistin resistance			1		1
*tetA*	Tetracycline resistance				1	
*sul1*	Sulphonamide resistance				1	
*sul2*	Sulphonamide resistance				1	
*bla*_TEM-1B_	Beta-lactam resistance				1	
Others						
*Iss*	Increased serum survival	1	1	1	1	1
*astA*	EAST-1 heat-stable toxin	1	1	1	1	1
*terC*	Tellurium ion resistance protein	1	1	1	1	1
*toxB*	Toxin B	1		1		1
*chuA*	Outer membrane hemin receptor	1	1	1	1	1
*ompT*	Outer membrane protease	1	1	1	1	1
*traT*	Outer membrane protein complement resistance	1	1	1	1	1
*mchF*	ABC transporter protein MchF				1	
*neuC*	Polysialic acid capsule biosynthesis protein	1	1	1	1	1
*sitA*	Iron transport protein				1	
*hlyF*	Hemolysin F				1	

**Table 4:** Phage sequences classified according to the PHASTER scores as intact, questionable, and incomplete >90, 70–90, <70, respectively.

Prophages	Type of Sequences			STEC O145 Strains				
	Intact	Incomplete	Questionable	1228	1229	1230	1231	1232
Entero_YYZ_2008_NC_011356		x x		x				x
Stx2_c_1717_NC_011357		x		x				
Entero_DE3_NC_042057		x x x x		x	x		x	x
Yersin_L_413C_NC_004745	x x x		x	x	x	x		x
Vibrio_12B8_NC_021073		x			x			
Entero_phi92_NC_023693		x x x			x	x	x	
Entero_VT2phi_272_NC_028656		x x x			x	x	x	
Entero_mEp460_NC_019716	x x				x		x	
Escher_P13374_NC_018846		x				x		
Entero_JSE_NC_012740		x					x	
Entero_WPhi_NC_005056		x					x	
Entero_P2_NC_001895	x						x	

The result of each strain is represented by a different color.

## Data Availability

Supporting data is available as part of the database of the Nation Reference Laboratory (Servicio Fisiopatogenia). Publicly archives datasets on: https://bancos.salud.gob.ar/recurso/boletin-integrado-de-vigilancia-n560-se-302021; https://bancos.salud.gob.ar/recurso/boletin-integrado-de-vigilancia-n329-se39-11102016 (accessed on 28 November 2021).

## Supplementary Materials

The following are available online at https://www.mdpi.com/article/10.3390/microorganisms10030582/s1, Figure S1: Dendrogram of STEC O145 representative strains of each pattern, Table S1: Outbreaks descriptions.
