# Supplementary material for: The Importance of Shiga Toxin-Producing Escherichia coli O145:NM[H28]/H28 Infections in Argentina, 1998–2020
Source: Microorganisms. 2022 Mar 7;10(3):582. doi: 10.3390/microorganisms10030582 (PMC8950694; doi:10.3390/microorganisms10030582)
Supplement: Supplementary file 1 [file microorganisms-10-00582-s001.zip › microorganisms-1454558-supplementary.pdf]

Article

The Importance of Shiga Toxin-Producing *Escherichia coli* O145:NM[H28]/H28 Infections in Argentina, 1998–2020

Claudia Carolina Carbonari [1,*], Elizabeth Sandra Miliwebsky [1], Gisela Zolezzi [1], Natalia Lorena Deza [1], Nahuel Fittipaldi [4], Eduardo Manfredi [1], Ariela Baschkier [1], Beatriz Alejandra D'Astek [1], Roberto Gustavo Melano [2,3], Carla Schesi [1], Marta Rivas [1] and Isabel Chinen [1]

[1] Servicio Fisiopatogenia, Departamento Bacteriología, Instituto Nacional de Enfermedades Infecciosas-ANLIS "Dr. Carlos G. Malbrán", City of Buenos Aires 1282, Argentina; emiliwebsky@anlis.gob.ar (E.S.M.); gzolezzi@anlis.gob.ar (G.Z.); ndeza@anlis.gob.ar (N.L.D.); emanfredi@anlis.gob.ar (E.M.); abaschkier@anlis.gob.ar (A.B.); badastek@gmail.com (B.A.D.); cschesi@anlis.gob.ar (C.S.); mrivas@inmunova.com (M.R.); ichinen@anlis.gob.ar (I.C.)

[2] Public Health Ontario, Toronto Laboratories, Toronto, Canada; roberto.melano@oahpp.ca (R.G.M.);

[3] Department of Laboratory Medicine and Pathobiology, University of Toronto, Toronto ON M5G 1M1, Canada

[4] Faculty of Veterinary Medicine, University of Montreal, Saint-Hyacinthe QC J2S 2M2, Canada; nfittipaldi@umontreal.ca

* Correspondence: ccarbonari@anlis.gob.ar

Supplementary Materials

Table S1. Outbreaks description. (†) Deceased case.

No. of Event	Type of Event	Index Case	Total No. of STEC Case & Contacts Detected	City, Province Date	PFGE Patterns of STEC O145 Strains Outbreak Associated
1	Institutional / day-care center outbreak	HUS case<br>1 year old boy<br>Co-infection<br>STEC shedding for 18 days	1 HUS case with long shedding. (Four strains STEC O145:NM[H28] *stx*2a/*eae*/*ehx*A and one STEC ONT:HNT)<br>1 HUS case and 1 asymptomatic case (O145:NM *stx*2a/*eae*/*ehx*A)	Rosario, Santa Fe<br>January, 2005	ARENMX01.0006 (2 HUS cases one present 2 strains) and ARENMX01.0043 (asymptomatic) with 88.9% similarity.<br>ARENMX01.0042 (2 strains from excretion) with 74% similarity.
2	Family outbreak	Undetermined	3 HUS cases<br>1 asymptomatic contact<br>(O145:NM[H28] *stx*2a/*eae*/*ehx*A)	Mar del Plata, Buenos Aires<br>January and February, 2006	ARENMX01.0061 - 100% similarity
3	Family outbreak	HUS case<br>20 months of age	1 HUS case (STEC negative) and 3 asymptomatic household contacts (2 sisters and the father)<br>(O145:NM[H28], *stx*2a/*eae*/*ehx*A)	Neuquén, Neuquén<br>November, 2006	ARENMX01.0006 - 100% similarity
4	Family outbreak	HUS case	1 HUS case and 1 asymptomatic contact (sister)<br>(O145:NM[H28] *stx*2a/*eae*/*ehx*A)	Córdoba, Córdoba<br>January 2011	ARENMX01.0040 - 100% similarity
5	Institutional / kindergarten outbreak	HUS case (†)<br>5 years old boy	1 HUS case (†), 1 1 BD case (3 positive samples) and 2 D non-complicated (one D with 3 positive samples)<br>(O145:NM[H28], stx_{2a}/*eae*/*ehx*A)<br>1 contact (O111:NM, stx_{1a}/*eae*/*ehx*A)	City of Buenos Aires<br>April, 2011	ARENMX01.0135 (HUS)<br>ARENMX01.0136 (BD 1°sample and D – 100% similarity)<br>ARENMX01.0137 (D 1st, 2nd and 3th sample; BD 2nd and 3thsample – 100%)<br>84.6% similarity among all the strains
6	Institutional / kindergarten outbreak	HUS case<br>Co-infection<br>10 months old girl	1 HUS (two positive samples) and 1 D case (O145:NM[28], stx_{2a}/*eae*/*ehx*A and O157:H7, stx_{2a}/stx_{2c}/eae/*ehx*A)<br>1 BD case (O157:H7, stx_{2a}/ stx_{2c} *eae*/*ehx*A)<br>1 Contact (O26:H11, stx_{1a}/*eae*/*ehx*A and O157:H7, stx_{2a}/stx_{2c}/ *eae*/*ehx*A)<br>1 D case (O91:H21, stx_{2c}/*ehx*A)<br>1 Contact (O103:NM, stx_{1a}, /*eae*/*ehx*A)	City of Buenos Aires<br>May, 2011	ARENMX01.0138 (HUS 1st and D – 100% similarity)<br>ARENMX01.0139<br>93.3% similarity among all the strains

			1 Contact (O22:H11, stx_{1a}/stx_{2c}/*ehx*A)		
7	Family out-break	HUS case 23 months old girl	1 HUS case and 2 contacts (father and sister) (O145:NM[H28], stx_{2a}/*eae*/*ehx*A)	City of Buenos Aires December, 2011	ARENMX01.0119 - 100% similarity
8	Family out-break	DS case 8 years old boy	1 BD case and 1 contact (sister) (O145:NM[28], stx_{2a}/*eae*/*ehx*A)	Tandil, Buenos Aires December, 2011	ARENMX01.0169 - 100% similarity
9	Institutional / Day-care center outbreak	HUS case 2 years old girl	1 HUS and 2 asymptomatic contacts (O145:NM[28], stx_{2a}/*eae*/*ehx*A)	San Carlos de Bariloche, Río Negro October, 2012	ARENMX01.0194 (HUS and contact – 100% similarity) ARENMX01.0212 (contact - 90.5% similarity)
10	Family out-break	HUS case	1 HUS and 1 asymptomatic contact (brother) (O145:NM[28], stx_{2a}/*eae*/*ehx*A)	City of Buenos Aires January 2015	ARENMX01.0207 – 100% similarity
11	Family out-break	HUS case 17 months old	1 HUS case (STEC negative) and 1 asymptomatic contact (sister) (O145:NM[28], stx_{2a}/*eae*/*ehx*A)	Villarino, Buenos Aires January 2016	ARENMX01.0274 (contact)
12	Family out-break	HUS case 8 months old boy	1 HUS case (STEC negative) and 1 asymptomatic contact (mother) (O145:NM[28], stx_{2a}/*eae*/*ehx*A)	San Carlos de Bariloche, Rio Negro February, 2016	ARENMX01.0261 - 100% similarity
13	Family out-break	BD case 8 years old boy	1 BD case and 3 asymptomatic contacts (2 sisters and 1 brother) (O145:NM[28], stx_{2a}/*eae*/*ehx*A)	Maisonave, La Pampa August, 2016	ARENMX01.0120 (BD 1st,$2^{nd}$and 4th – 100% similarity) and ARENMX01.0279 (contacts) with 92.4% similarity
14	Family out-break	D case 1 year old boy	1 D case and 6 contacts with diarrhea (4 brothers, 2 sisters) (O145:NM[28], stx_{2a}/*eae*/*ehx*A)	Gral. Pico, La Pampa December, 2016	ARENMX01.0281 - 100% similarity
15	Institutional / Kindergarten outbreak	HUS case 5 years old girl	3 HUS cases (two are brother and sister) (O145:NM[28], stx_{2a}/*eae*/*ehx*A)	Vicente López, Buenos Aires July, 2017	ARENMX01.0303 – 100% similarity
16	Family out-break	D case 3 years old boy	1 D case and 2 asymptomatic contacts (mother and unknown) (O145:NM[28], stx_{2a}/*eae*/*ehx*A)	San Justo, Buenos Aires February, 2018	ARENMX01.0120 (D 1st, 2nd and contacts – 100% similarity)
17	Family out-break	HUS case 20 months old boy	1 HUS case and 2 asymptomatic contacts (sister and mother) (O145:NM[28], stx_{2a}/*eae*/*ehx*A)	Hilario Lagos, La Pampa March, 2018	ARENMX01.0319 (HUS 1st, 2nd, 3th and contacts – 100 similarity)
18	Family out-break	HUS case 6 months old boy	1 HUS and 1 asymptomatic contact (father) (O145:NM[28], stx_{2a}/*eae*/*ehx*A)	Bahía Blanca, Buenos Aires November 2018	ARENMX01.0340 – 100% similarity
19	Family out-break	HUS case 2 years old	1 HUS (STEC negative) and 1 asymptomatic contact (mother) (O145:NM[28], stx_{2a}/*eae*/*ehx*A)	Valcheta, Rio Negro May 2018	ARENMX01.0324
20	Family out-break	HUS case 2 years old	1 HUS (STEC negative) and 2 asymptomatic contacts (sister and mother) (O145:NM[28], stx_{2a}/*eae*/*ehx*A)	Bahia Blanca, Buenos Aires March 2019	ARENMX01.0076 – 100% similarity
21	Family out-break	D case 12 years old boy Long shedding (5 positive samples)	1 D case and 1 asymptomatic contact (father) (O145:NM[28], stx_{2a}/*eae*/*ehx*A)	San Carlos de Bariloche, Rio Negro June 2019	ARENMX01.0399 (2nd) ARENMX01.0401 (3th) ARENMX01.0402 (4th, 5th and 6th sample) with 94.3% similarity (Contact non-typeable)
22	Family out-break	D case 2 years old girl	2 D case (index case and sister) (O145:NM[28], stx_{2a}/*eae*/*ehx*A)	Realicó, La Pampa November 2020	ARENMX01.0400 (HUS 1st, 2nd and contact – 100% similarity)
