# Supplementary material for: The Importance of Shiga Toxin-Producing Escherichia coli O145:NM[H28]/H28 Infections in Argentina, 1998–2020
Source: Microorganisms. 2022 Mar 7;10(3):582. doi: 10.3390/microorganisms10030582 (PMC8950694; doi:10.3390/microorganisms10030582)
Supplement: Supplementary file 1 [file microorganisms-10-00582-s001.zip › microorganisms-1454558-supplementary-FigureS1.pptx]

## Slide 1
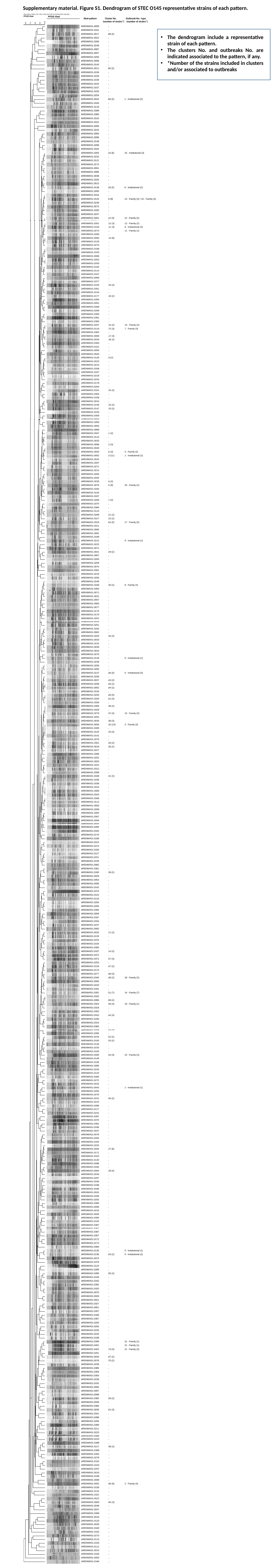

Supplementary material. Figure S1. Dendrogram of STEC O145 representative strains of each pattern.
XbaI-pattern Cluster No. Outbreak No.- type
 (number of strains*) (number of strains*)
The dendrogram include a representative strain of each pattern.
The clusters No. and outbreaks No. are indicated associated to the pattern, if any.
*Number of the strains included in clusters and/or associated to outbreaks
